# Ten simple rules to create biological network figures for communication

**DOI:** 10.1371/journal.pcbi.1007244

**Published:** 2019-09-26

**Authors:** G. Elisabeta Marai, Bruno Pinaud, Katja Bühler, Alexander Lex, John H. Morris

**Affiliations:** 1 Electronic Visualization Laboratory, University of Illinois at Chicago, Chicago, Illinois, United States of America; 2 Laboratoire Bordelais de Recherche en Informatique, University of Bordeaux, Bordeaux, France; 3 Biomedical Image Informatics Department, VRVis Research Center, Vienna, Austria; 4 Department of Computer Science, University of Utah, Salt Lake City, Utah, United States of America; 5 Department of Pharmaceutical Chemistry, University of California San Francisco, San Francisco, California, United States of America; Whitehead Institute for Biomedical Research, UNITED STATES

## Abstract

Biological network figures are ubiquitous in the biology and medical literature. On the one hand, a good network figure can quickly provide information about the nature and degree of interactions between items and enable inferences about the reason for those interactions. On the other hand, good network figures are difficult to create. In this paper, we outline 10 simple rules for creating biological network figures for communication, from choosing layouts, to applying color or other channels to show attributes, to the use of layering and separation. These rules are accompanied by illustrative examples. We also provide a concise set of references and additional resources for each rule.

## Author summary

Biological network figures are ubiquitous in the biology and medical literature. In this paper, we outline 10 simple rules for creating biological network figures for communication, from choosing layouts, to applying color or other channels to show attributes, to the use of layering and separation.

## Introduction

Biological networks are present in many areas of biology, including studies of cancer and other diseases, metagenomics, pathway analysis, proteomics, molecular interactions, cell–cell interactions, epidemiology, network rewiring due to perturbations or evolution, etc. Increasingly, published studies in these areas and many others include figures meant to convey the results of one or more experiments or of the network analysis carried out. As a result, biological network figures are ubiquitous in the biology and medical literature. On the one hand, a good network figure is able to quickly provide information about interactions between items and can often convey the nature and degree of interactions, as well as enable inferences about the reason for those interactions. On the other hand, good network figures are difficult to create. The scale of data can often obscure the relationships that the figure is trying to convey, the spatial layout and distribution of the network can be difficult to interpret, and the many ways in which data can be mapped onto network representations provide an easy pathway to violating best practices of data visualization.

Some relatively simple rules, when followed, can significantly improve the likelihood that a network visualization will "tell the story" the author intends. The following set of rules was a result of a week-long seminar that brought together leading biology, bioinformatics, and visualization researchers from different countries [1]. Note that the rules we give are meant for static figures as used for publications, not for dynamic figures or for interactive or exploratory tools that allow users to manipulate the data view. The rules are tightly interconnected and, in general, follow the typical visualization design decision process (without forming a decision tree, due to their interconnectedness), from determining first the intended message of the illustration we seek to create [2, 3] to selecting appropriate encodings for that message and network. In order to provide a useful interpretation of these rules, we use real data for our illustrations below, and in many cases, we utilize network figures from the bioinformatics literature. In no way do we mean to detract from the science or experimental results that these published figures are trying to represent. As already noted, good network figures are difficult to create, and even some of the figures we use to illustrate specific rules below may come up short with respect to another rule. Last but not least, for each rule we also provide a concise set of references and resources where the interested reader may find additional information on the topic.

## Rule 1: First, determine the figure purpose and assess the network

The first rule is also arguably the most important: Before creating an illustration, we need to establish its purpose [4] and then the network characteristics. When establishing the purpose, it helps to first write down the explanation (caption) we wish to convey through the figure and note whether the explanation relates to the whole network; to a node subset in the network; to a temporal, causal, or functional aspect of the network; to the topology of the network; or to some other aspect. This analysis needs to happen before we draw the network because the data included in the view, the focus of the figure, and the sequence we use to visually encode the network should support the explanation that we wish to convey. For example, salient aspects of the figure may need to be displayed centrally, in larger size, or marked by annotations. Second, we need to assess the network in terms of scale, data type, structure, etc. These network characteristics will further constrain salient aspects of the visualization, such as the color, the shape, the marks used, and the layout of the network [5].

**Fig 1:**
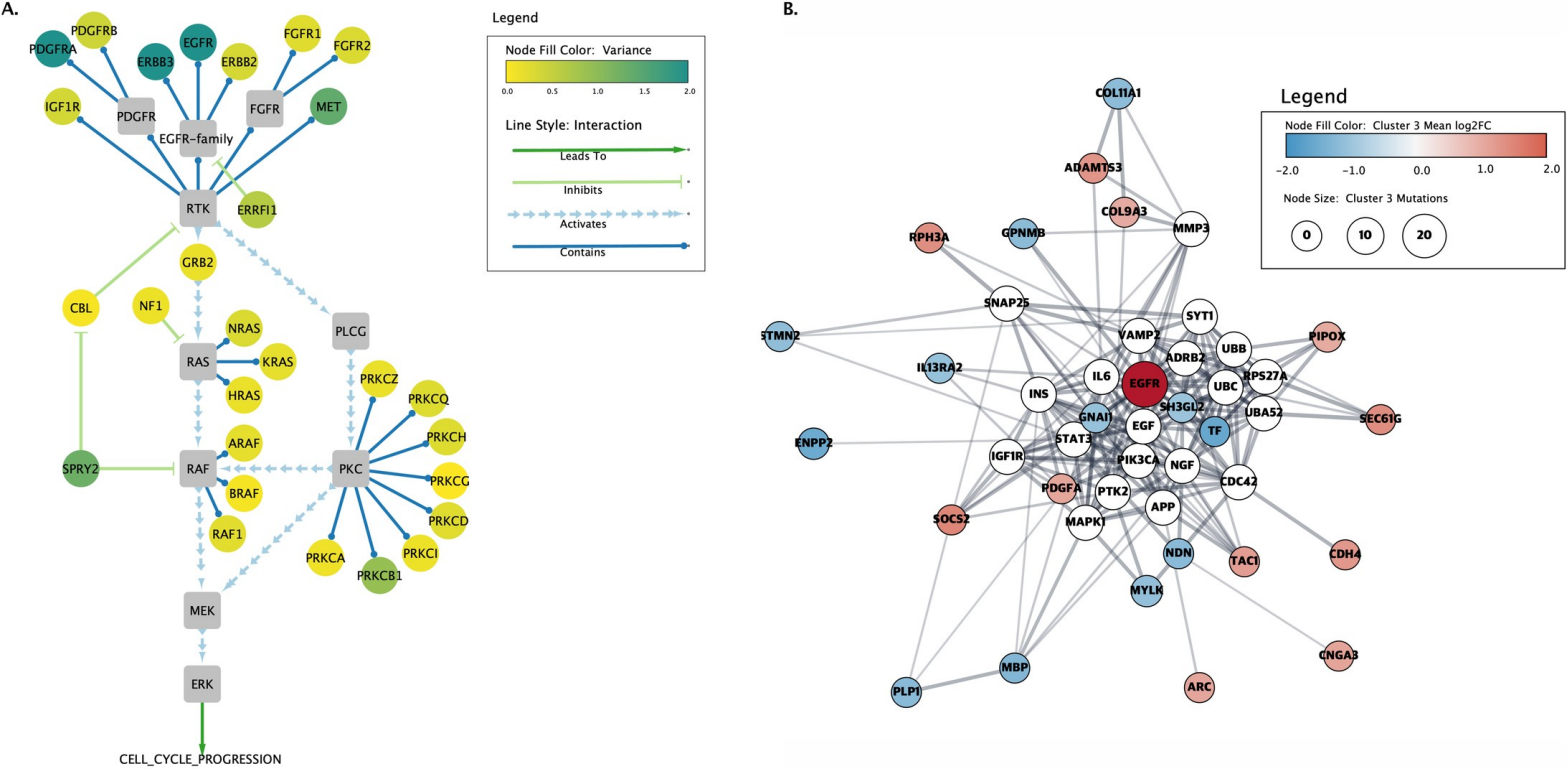
First, determine the figure purpose and assess the network. Two representations of proteins involved in GBM. The left image (A) shows a curated cancer signaling pathway taken from the TCGA's original Mondrian plugin to Cytoscape (Cytoscape Consortium; https://cytoscape.org/). The node color represents the overall variance of expression across a set of patients, and the lines and arrows represent the function of the interactions between the proteins. In the right image (B), a PPI network was created using the Cytoscape stringApp and annotated with data downloaded from TCGA. The colors represent the fold change for subtype 3 of GBM, the node sizes vary with the number of mutations, and the edges represent functional associations. GBM, glioblastoma multiforme; PPI, protein–protein interaction.

Fig 1 delivers two messages about proteins known to be involved in glioblastoma multiforme (GBM). The first figure is a RAS signaling cascade in a curated GBM network. Because the message of the figure relates to protein interaction functions, the figure uses a data flow encoding, with nodes connected by arrows. The nodes are colored by the expression variance across samples. The second figure is a STRING protein–protein interaction (PPI) network representing proteins that show significant expression changes in subtype 3 of GBM, in addition to 20 additional proteins to improve connectivity; the colors represent the fold change, and the size represents the number of mutations. Because the message of this figure relates to the structure of the network, not its functionality, the nodes are connected by undirected edges, and the nodes are placed to reinforce the structure. Furthermore, note how the quantitative color scheme (yellow to green gradations) in the first network shows expression variance, whereas the divergent color scheme (red to blue) in the second network emphasizes the extreme values of differential expression for one GBM subtype. Similarly, the edges in the first network are arrows indicating function, whereas in the second network, they are edges to indicate structure. Each image tells a different story: The first message is about network functionality, the second about the network structure.

## Rule 2: Consider alternative layouts

**Fig 2:**
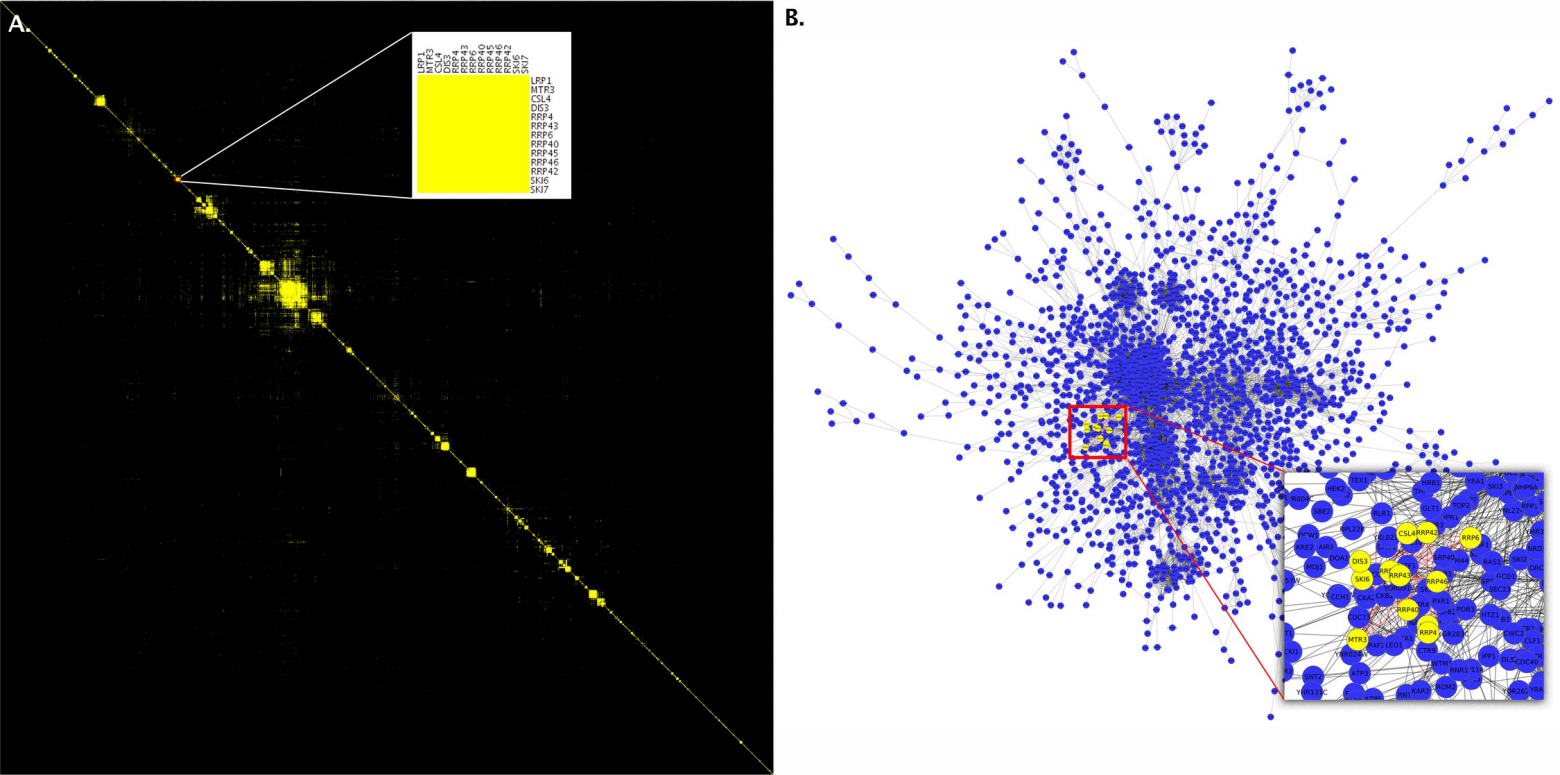
Consider alternative layouts. These two images represent the same data from Collins and colleagues [6]. The image on the left (A) shows an adjacency matrix representation of the network. The inset within the image shows a cluster identified on the diagonal that represents the exosome complex. The image on the right (B) is of the same data depicted as a node-link diagram with the same nodes highlighted. Notice how difficult it is to see the close interaction between the nodes, even in the inset in this second image, due to the clutter resulting from other nodes. These images were produced in Cytoscape (Cytoscape Consortium; https://cytoscape.org/) with the clusterMaker2 app and postprocessed in Photoshop (Adobe; https://wwww.adobe.com/) to merge in the insets.

Node-link diagrams are the most common way to display network data. Node-link diagrams are familiar to readers, and they can show relationships between nodes that are not immediate neighbors. However, node-link diagrams also have drawbacks: For dense and large networks, they tend to produce significant clutter, edge attributes are difficult to visualize, and node labels often cause even more clutter. An alternative network representation is adjacency matrices (see Fig 2). An adjacency matrix lists all nodes of a network horizontally and vertically. An edge is represented by a filled cell at the intersection of the connected nodes.

Adjacency matrices have several advantages: First, they are well suited for dense networks with many edges, as every possible edge is represented by a cell [7]. Second, they can encode edge attributes, for example, with color or color saturation of a cell. Third, adjacency matrices excel at showing neighborhoods of nodes and clusters, provided the node order is optimized [8]. Fourth, the layout of the matrix makes it easy to display readable node labels, whereas labels in a comparable node-link layout would cause significant clutter. Matrix layouts are easy to implement, e.g., in R, Python, or JavaScript, even without dedicated graph visualization libraries. In practice, using an appropriate column/row reordering algorithm is crucial [8].

Another alternative to traditional node-link layouts is fixed layouts: Here, the nodes are positioned such that the position of the nodes themselves encodes data. A common example is networks shown on top of maps, or links on top of linear or circular layouts, such as is commonly used for genomic data visualization in Circos [9]. Finally, when the graph to be shown is a tree, we can also make use of implicit layouts, such as icicle plots [10], sunburst plots [11, 12], or treemaps [13, 14]. Implicit layouts encode the relationships between parents and children by adjacency, and the size of the leaves is commonly scaled according to an attribute. S. Ribecca's Data Visualisation Catalogue (datavizcatalogue.com) provides a wide although nonexhaustive array of possible representations.

## Rule 3: Beware of unintended spatial interpretations

Node-link diagrams map nodes to locations in space. In turn, Gestalt theory (in particular, the principles of grouping) teaches us that the spatial arrangement of nodes and edges influences the reader’s perception of the network information—even if there is no meaning [4]. Thus, the right layout can effectively enhance features and relations of interest, but the wrong layout might easily lead to misinterpretation. An example of such a misinterpretation can be found in the Atlas of Science [16]. Although aesthetically pleasing, the node-link diagram shows a defective spatial encoding that suggests a black hole of knowledge.

**Fig 3:**
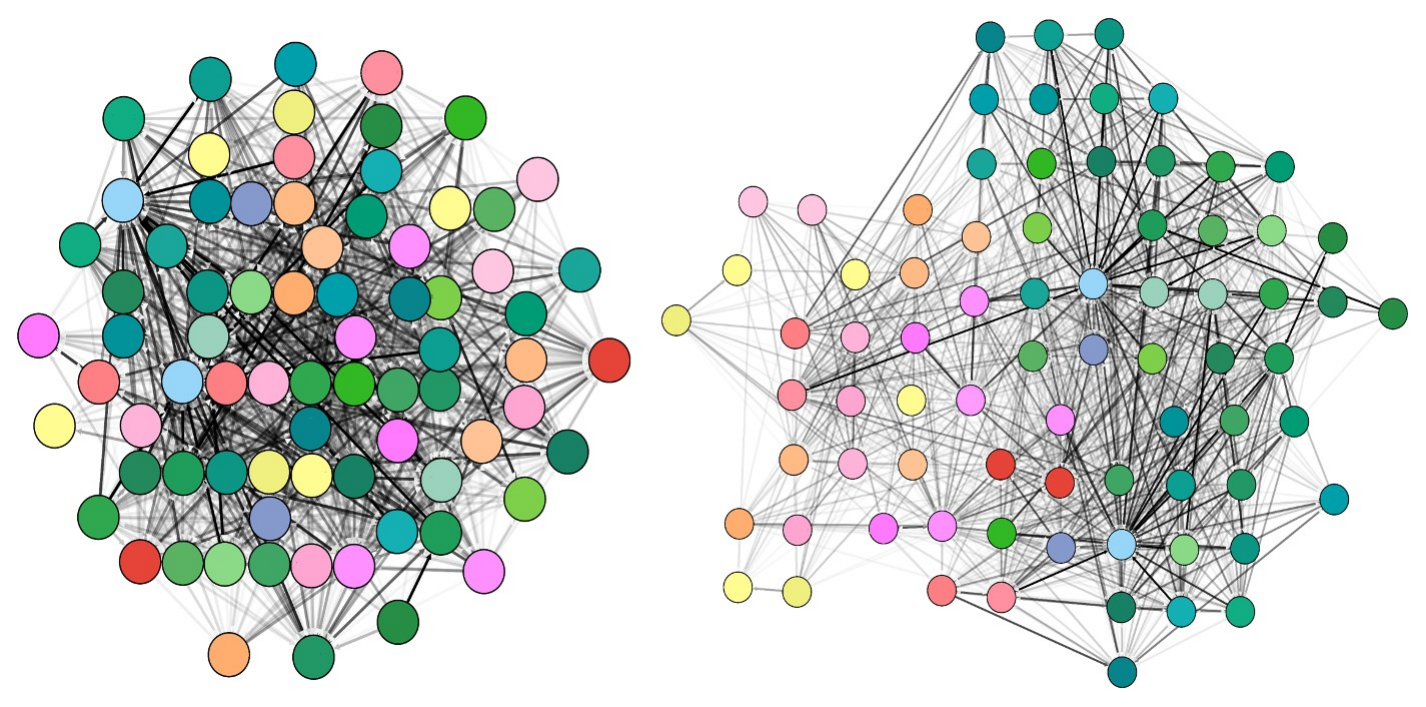
Beware of unintended spatial interpretations. This figure shows two illustrations representing the same region of the normalized structural mouse brain connectivity data set described by Ganglberger and colleagues [17]. The data are derived from the Allen Mouse Brain Connectome dataset [18]. The illustrations have been generated using the Cytoscape.js (Cytoscape Consortium; https://cytoscape.org/) implementation of the force-directed layout algorithm CoSE. The left image uses connectivity strength as the driving force for the layout, posing strongly connected nodes closely together, but at the same time neglecting the spatial context of the network. Instead, the second layout in the right image is driven by the spatial relation of brain regions, generating automatically a "flattened" mouse brain representation as seen from above. Symmetry and spatial positions are approximately reproduced. Structural connectivity strength is encoded by the gray-level color scale of the edges. CoSE, compound spring embedder.

Proximity, centrality, and direction of node arrangement are the most prominent principles to be considered when integrating spatiality into meaningful network representations: Nodes drawn in proximity will be interpreted as conceptually related; nodes grouped together are also perceived as more similar to each other than nodes outside the group. We may use as a similarity measure the connectivity strength between two nodes (an edge-based measure), similarity of the content carried by the nodes, e.g., nodes being part of the same brain region or conceptual group (a node-based measure), or a mixture of both. This measure is then used as an optimization criterion for the layout algorithm (Fig 3). Most prominent layouts are force directed and interpret the given similarity measure as an attracting force for nodes, whereas graph layouts based on multidimensional scaling perform better for cluster detection [15]. Centrality is a design principle in which the center and periphery may represent metaphorically high relevance and secondary relevance, respectively. A layout may be spatially constrained to display the focus of the illustration in the center of the figure. The third design principle is direction: The vertical dimension represents power, from light/good (up) to heavy/bad (down) and also flow of information or development (up to down) or in the horizontal direction (left to right in Western cultures).

Most open-source network drawing tools like Cytoscape (Cytoscape Consortium; https://cytoscape.org/) and yEd (yWorks GmbH; https://www.yworks.com) provide a rich selection of different layout algorithms. Beside these resources, drawing networks and developing appropriate layout methods is a whole scientific discipline by itself. An excellent source for diving deeper into the world of graph drawing algorithms is http://graphdrawing.org/.

## Rule 4: Provide readable labels and captions

**Fig 4:**
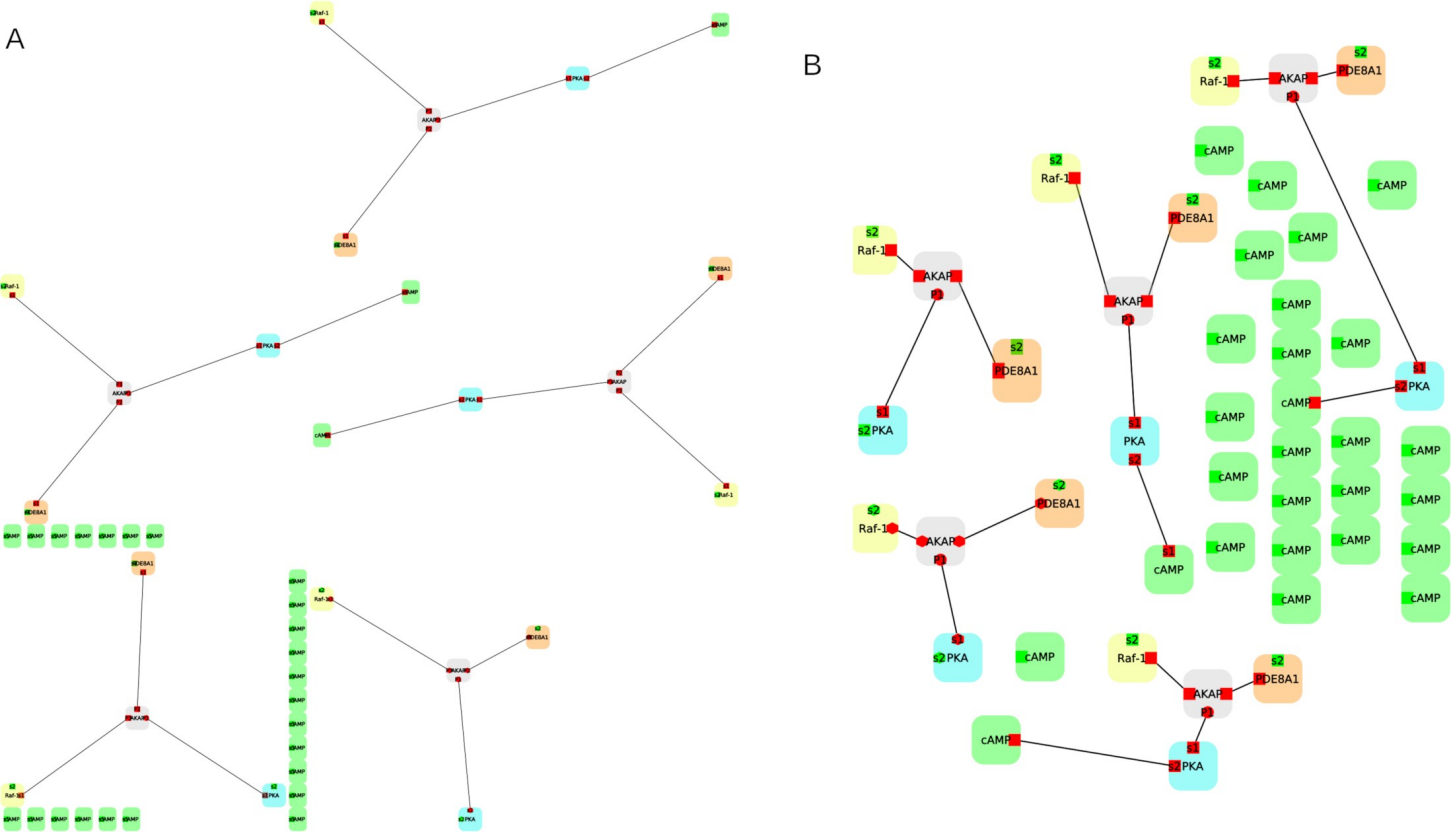
Provide readable labels and captions. (A) An example network based on PPI data from Andrei and colleagues [34], in which the node labels are too small to be legible. (B) The same network, but this time the layout has been improved to make better use of the available space, resulting in larger labels. The two images have been generated using the open-source software Porgy (http://porgy.labri.fr). PPI, protein–protein interaction.

The proper use of labels and captions can help explain and clarify the icons, colors, and visual representations present in a network figure. First, network labels and, in general, text in a network figure have to be legible. To be legible, labels in the figure should use the same (or larger) font size as the caption font, not smaller. Fig 4A shows PPI data from Andrei and colleagues [34], in which the node labels are too small to be legible. In Fig 4B, the layout has been modified to make better use of the available space, resulting in larger labels. Although this type of manipulation may not always be possible (for example, Fig 10 in Wenskovitch and colleagues [19] shows the similarity among 4 large-scale network models with no room for larger labels), in such cases, one should at least provide an online high-resolution version of the network that can be zoomed in. Furthermore, whereas it is tempting to rotate text affiliated with specific network elements in order to optimize space, all network text should use a horizontal orientation: Vertical or tilted text is hard to read. To be legible, all text should also have good contrast with the background, preferably black on white or white on black.

The figure and its caption (the brief explanation appended to an image) should each be able to stand on their own and provide both context and interpretation. The caption, in particular, should tell the reader what to notice in the network figure, without the reader needing to chase the figure reference in the manuscript text. The network figure text should further clarify the meaning of all unusual visual markers and channels used in the network representation, including all colormaps. Last but not least, labels should be properly placed within the network figure. For example, inset and subfigure labels should be placed in clear proximity to that element. Whenever possible (i.e., when the figure is not too cluttered), use direct labeling instead of numerical pointers to a legend; numerical pointers place a higher cognitive load on the reader.

## Rule 5: Choose the right level of detail

Depending on the intended meaning of a figure, it may be beneficial to show fewer details, even if they are relevant, in order to bring into better focus the item(s) or relationship(s) of interest (reference [5], Chapter 13). The level of detail shown can also change locally across the figure. If, for example, one is interested in showing centrally the details of a network, there is no need to display the data at the periphery with the same (high) level of detail. To keep the context of the visualization clear, the entire structure can be shown in an aggregated form, around the item of interest. Aggregation can be performed at the level of items, based on dimensionality reduction over the item attributes (e.g., principal component analysis), or based, for example, on a spatial aggregation of geo-collocated items into groups. Aggregation may also be performed at the level of relationships, via, e.g., edge bundling algorithms. The wise use of aggregation in combination with a variety of visual marks and channels can significantly reduce visual clutter.

**Fig 5:**
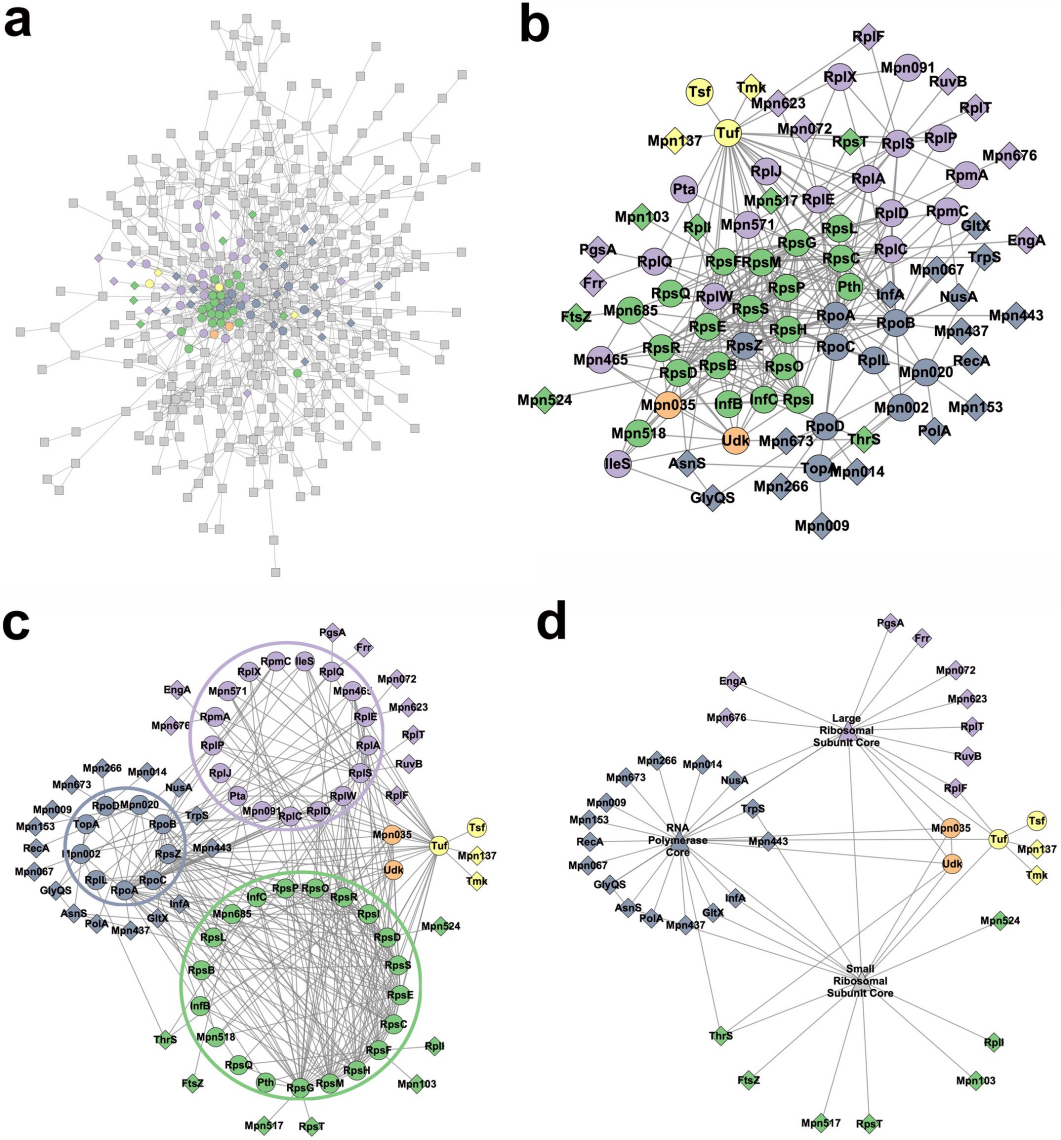
Choose the right level of detail. Example aggregation using data from Kuhner and colleagues [21], which replicates the sequence of steps described in Gehlenborg and colleagues [20], from a hardly readable network (A), gradually through (B) and (C), to a legible, aggregated version of the same network (D).

Fig 5 shows images made with Cytoscape of protein interaction data with 5 complexes (computationally determined) colored using data from Kuhner and colleagues [21]. This figure replicates the sequence of steps described in Gehlenborg and colleagues [20]. Network *a* is the original protein interaction network (> 400 proteins). According to Gehlenborg and colleagues, this first network is hardly readable, and nothing really interesting is visible. Network *b* is a recomputed network after removing nodes not of interest. Clusters based on the complexes’ color start to emerge. Network *c* is a manual refinement to emphasize the structure of protein complexes and the interactions between them. Finally, network *d* proposes to collapse nodes in each complex core (e.g., nodes inside each colored circle are replaced by only one triangle of the same color) to simplify the network and emphasize global properties, which is the aim of the figure.

## Rule 6: Use color responsibly

Color is a complex topic [23], and here, we touch only on the aspects most relevant to bionetwork visualization. Color is a perception and not visible electromagnetic radiation (light waves are not colored): Most, but not all, people experience the sensation "blue" with wavelengths near 400 nm. The color humans perceive depends on the eye–brain mechanism, and therefore color perception is influenced by context, training, or abnormalities such as color blindness, which affects 8% of the male population and often results in an inability to distinguish red from green. For this reason, red–green color encodings of network data should be avoided. Human vision is also much more sensitive to slight changes in the luminance of a color (its intensity or value) than slight changes in the quality of a color (its hue and saturation) [24]. Therefore, it is a good idea to convert the network figure to grayscale and make sure that the information encoded in the diagram is still legible. In a nutshell, get the figure right in grayscale first. In terms of saturation, areas of saturated color draw attention and are best used on small areas such as nodes; use saturated colors sparingly and to draw attention. The hue component (the color quality that distinguishes red, green, blue, etc.) is also powerful: Hue families can code related items. Qualitative maps (i.e., multihued maps) should be used only for categorical coding, to indicate different qualities or identities of data. Because humans have no sense of whether blue is more or less than orange, to encode ordinal data, figures should use a progression of luminance values, similar to topographic maps. All else being equal, blue-family hues tend to recede, whereas warmer red-family hues tend to come forward, and so the use of these two families together in a network may result in an unwanted 3D effect [25]. Transparency can be further used to modulate a color: Transparent markers tend to be perceived as being in the background.

**Fig 6:**
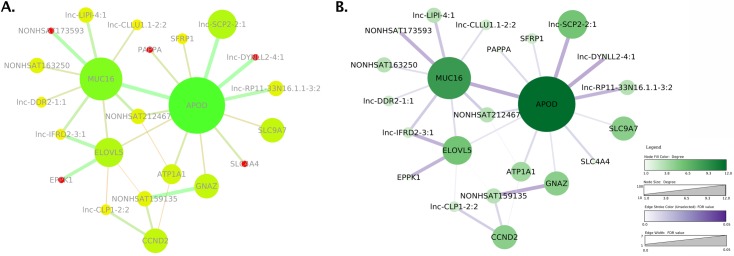
Use color responsibly. Two network images based on data from Khaled and colleagues [22]. (A) is a recreation of the original Fig 3B shown in the paper, including the color-blind and saturated color scheme, which makes it difficult to perceive the relative importance of the nodes. The colormap also groups unrelated edges and nodes together through similar colors, whereas the node labels in light gray have low luminance contrast with the white background and are difficult to read. (B) shows an improved version, including a legend and appropriate and separate quantitative colormaps for edges and nodes. Both images were created with Cytoscape (Cytoscape Consortium; https://cytoscape.org/) and postprocessed using Photoshop (Adobe; https://www.adobe.com/) to assemble them.

In Fig 6A, the colormap encodes the node degree using a two-tailed gradient (saturated yellow to saturated green) and saturated red for 1. The color scheme is not color-blind safe and employs saturation incorrectly. Some edges use, confusingly, the same hue as some unconnected nodes. The gray figure text has also poor contrast with the background (i.e., the text and background have similar luminance), making it hard to read. The revised image in Fig 6B uses a ColorBrewer (http://www.colorbrewer2.org) sequential colormap for the node degree, a separate sequential colormap for edges, and black figure text. The result is a significantly clearer figure, although the text contrast with colored backgrounds could be further improved.

## Rule 7: Use other visual marks and channels appropriately

**Fig 7:**
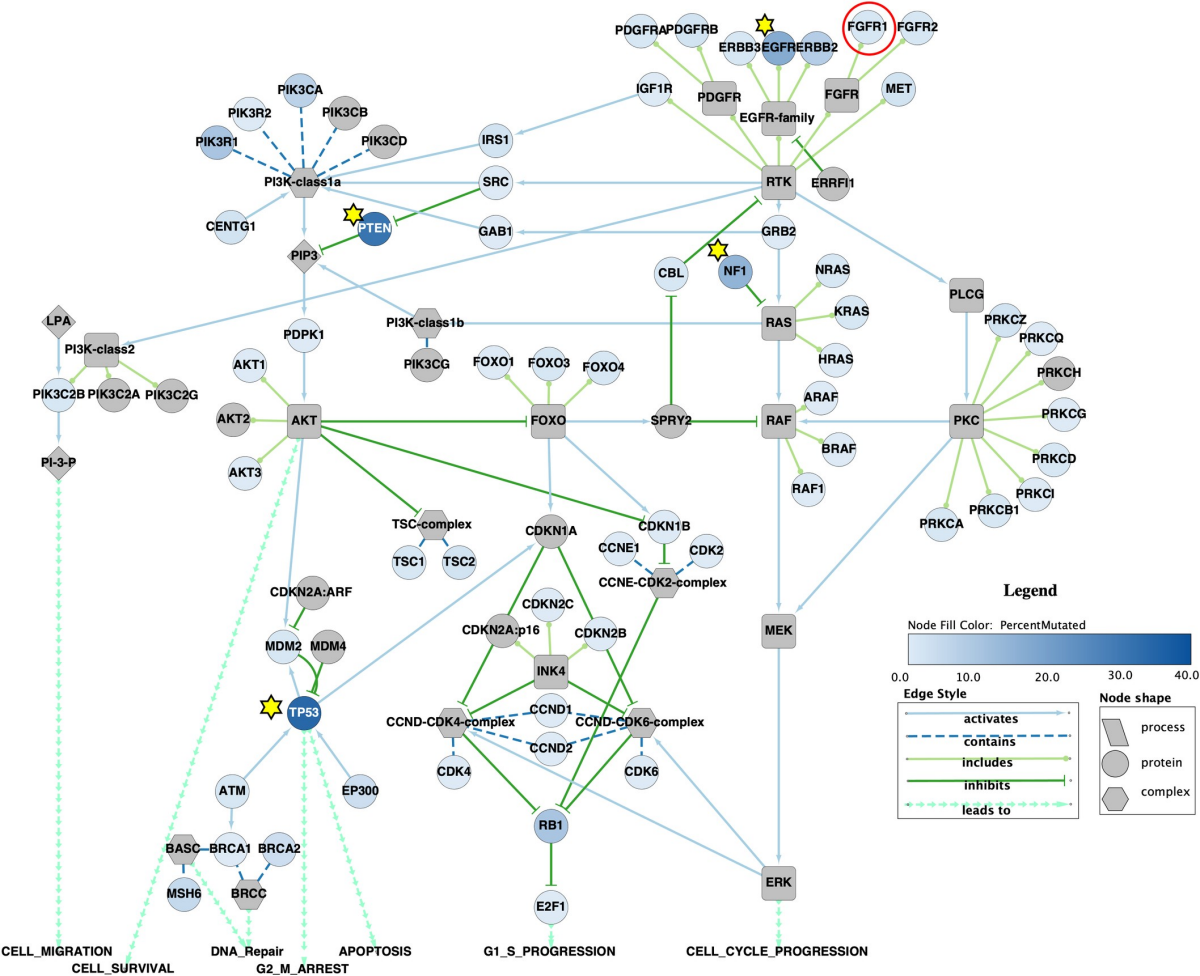
Use other visual marks and channels appropriately. In this Cytoscape (Cytoscape Consortium; https://cytoscape.org/) recreation of Fig 3 from Morris and colleagues [26], the authors used several different marks to explain the data in the network, including stars to indicate highly mutated nodes (in addition to the color gradient) and a red circle to indicate the subject of one of the scenarios outlined in the paper. The authors also used different node shapes to distinguish among complexes, proteins, and processes, and different line and line ending styles to indicate the relationship among the nodes.

Whereas color is incredibly powerful, other visual marks and channels are also important. Marks are basic geometric elements that depict items or links, whereas channels control the appearance of marks. Marks can be, with increasing dimensionality, dots, lines, arrows, blobs or polygons (marks with area) or volumetric glyphs (marks with volume). Some channels are position (see Rule 4), color (see Rule 6), shape, size, tilt, area, and volume. Using a variety of marks wisely can create more powerful displays, through increased flexibility, and further allows layering and separation of information for more effective displays (Rule 8). With respect to marks, in general, dots and glyphs represent items, whereas lines and arrows represent relationships between items. Blobs represent regions or containers of items. Arrows are asymmetric lines that represent asymmetric relations and can change drastically the meaning of a figure: diagrams with arrows tend to be interpreted as functional, presenting a sequence of actions and outcomes. In contrast, diagrams without arrows tend to be interpreted as structural, specifying the location of parts relative to one another [4]. With respect to channels, position, color, and shape are identity channels, which means that a set of shapes can be used to distinguish different categories and so can a set of colors or a set of predefined positions [5]. The remaining channels are magnitude or quantitative channels, which means that a set of sizes (small, medium, large, etc., or weak, medium, strong, etc.) can be used to distinguish different quantities or attribute strength of a specific category, and so on. The example in Fig 7 shows network data from Morris and colleagues [26] and makes effective use of multiple visual marks and channels.

## Rule 8: Use layering and separation

The goal of any figure is to communicate information. Communication can be difficult if the key information is obscured by too much “clutter.” We can raise the prominence of key information by imagining that different classes of information belong in different layers and that the key information is sitting on a higher layer in the figure and by providing visual separation between the layers. Once we decide on how we would like the information organized, layering and separation [27] are traditionally accomplished by means of assigning a specific weight, color, opacity, or size to each layer of information although we can also use spatial cues such as grouping to highlight relationships. For example, we can decrease the weight, luminance, saturation, opacity, or size of less important information, and increase the weight, luminance, saturation, opacity, or size of the key information to make it more visually salient.

**Fig 8:**
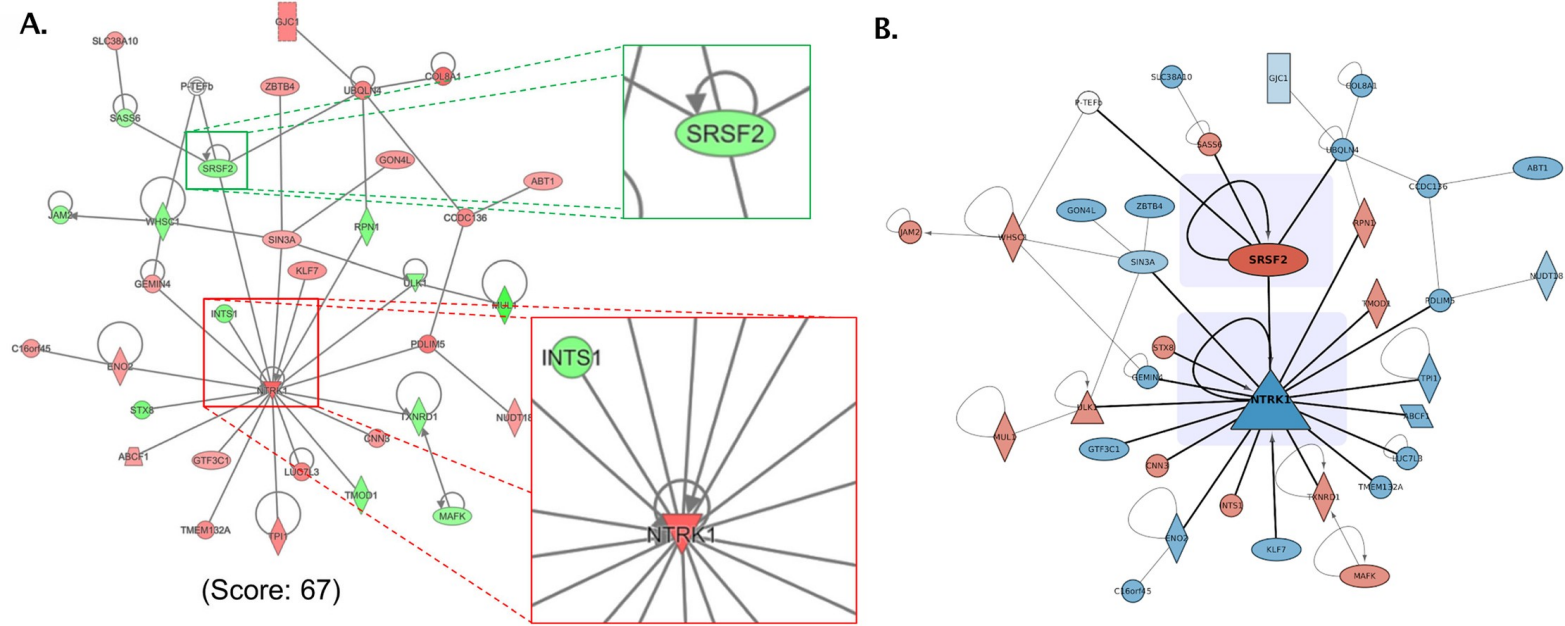
Use layering and separation. (A) Reconstruction of Fig 5A from Preston and colleagues [28], which contains the largest subnetwork resulting from a pathway and enrichment analysis. Callouts call attention to the neighborhoods around SRSF2 and NTRK1. (B) Modified image after changing the color scheme to avoid color-blind issues, decreasing the weight of the edges that do not connect to the key nodes and increasing the size of the key nodes. Nonkey nodes and self-edges were also de-emphasized by making them slightly transparent. Subtle shading behind the two key nodes was applied to provide additional separation.

As an example, consider the images in Fig 8. The left image is a reconstruction of Fig 5A from Preston and colleagues [28], showing the largest subnetwork resulting from a pathway and enrichment analysis. Based on the callouts, the key data the authors want to convey are the neighborhoods around SRSF2 and NTRK1. The image on the right is an improved version in which we decreased the weight of those edges that do not connect to the key nodes and increased the size of key nodes (Rule 7). Nonkey nodes and self-edges were also rendered transparent, which effectively leads to a perception of these nodes and edges being in the background (Rule 6). Typically, if self-edges are not germane to the point being made by the image, they would be removed. Last but not least, subtle shading behind the two key nodes was applied to provide additional separation.

## Rule 9: Use multiple figures

**Fig 9:**
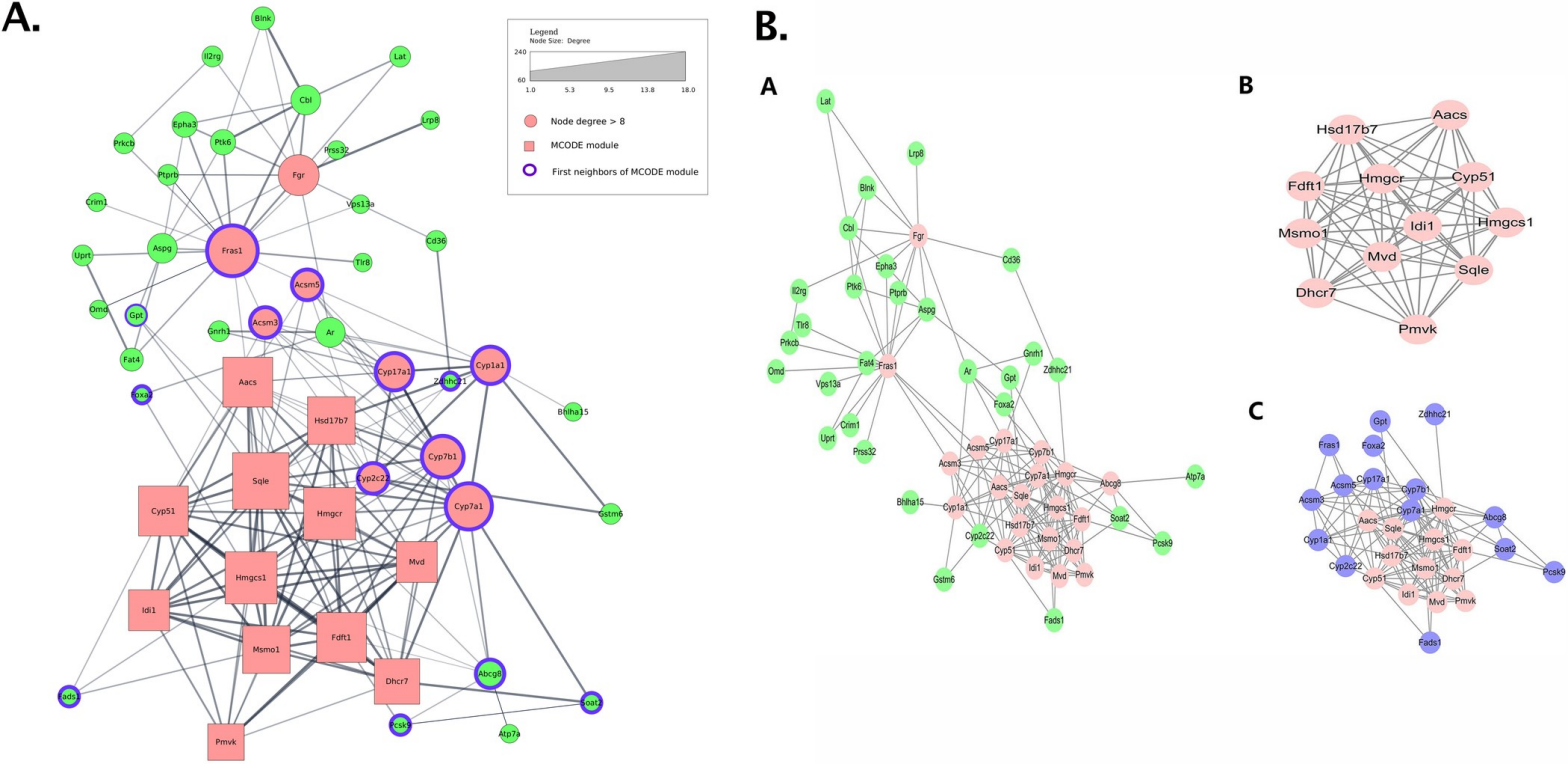
Use multiple figures. (A) An image constructed from the data provided by Zhu and colleagues [29] but constrained to show everything in a single view. The result is a very confusing image, and, from the viewer’s perspective, it is hard to determine what is important. (B) The original image from Zhu and colleagues. Fig 5, where the authors split the network into 3 views, each view with a different focus. The first view (A) highlights the high degree nodes, the second view (B) shows the MCODE component, and the third view (C) adds the first neighbors to that component. MCODE, molecular complex detection algorithm.

Another kind of clutter in a network figure happens when there is too much information vying for the attention of the viewer. Under these circumstances, it is often better to split that information into multiple figures, each emphasizing a different point. Multiple figures can also effectively illustrate a sequence in the illustration. Thus, as a rule of thumb, count the number of visual properties an image uses to map data. If it is greater than 3, and they are not redundant (i.e., not intentionally mapping the same value for emphasis) and their interaction is not the point being made (i.e., overexpressed genes are also hubs), think about separating the image into multiple separate figures, each one emphasizing a different point and potentially focusing on relevant subnetworks. Another interesting aspect is the use of one image (e.g., A in Fig 9B) to provide overall context for the visualization of subnetworks. This overview + detail approach can be very useful. However, an extremely dense network with many overlapping nodes will not provide effective overview or context. Alternative models to the "overview-first" paradigm [30] include a "search-first" paradigm [31] and a "details-first" paradigm [32], depending on the interests and background of the target audience.

As an example, Fig 9A shows an image constructed from the data provided by Zhu and colleagues [29]. The "overview" network (A) is itself a 51-node subnetwork of the full 195-node network that the authors initially queried. This image includes several different pieces of information: The node colors indicate whether the node is a hub, square nodes represent a cluster found by the molecular complex detection algorithm (MCODE), and the purple borders indicate the first neighbors of that cluster. The result is a confusing image, in which it is hard to determine what is important—the information does not rise above the clutter. Now, consider Fig 9B, which was the image the authors used. They split the network into three views. The first figure uses color to show degree, and it also provides an overall context for the subnetworks. The second network shows the results of the MCODE algorithm, and the third network shows those nodes plus their first neighbors. In each case, it is much easier to determine the point of the image.

## Rule 10: Do not use unjustified 3D

Many people think that if two dimensions (2D) are good, three dimensions (3D) must be better. As the printed medium evolves, video recordings and interactive displays, including virtual reality technologies, also become of interest. However, in the context of biological network displays, it is important to be aware that depth has important differences from the other two planar dimensions. 3D is seldom appropriate for such displays, due to documented issues related to depth perception inaccuracies, occlusion, perspective distortion, and so on (reference [5], Chapter 3). 3D is easy to justify when the users’ tasks involve 3D shape understanding, for example, in molecular structures, which inherently have spatial structures. In such cases, the benefits of 3D absolutely outweigh the perception costs, and designers are justified in investing in interaction idioms designed to mitigate such costs. For example, occlusion hides information—some objects cannot be visible because they are hidden behind other objects. Even though the occluded nodes can be discovered via interactive navigation, the navigation has a time and cognitive cost. Occlusion can be also mitigated through the use of motion parallax (motion cues) [33], which also has an associated cost. In all other contexts, using 3D needs to be carefully justified in the context of the higher cognitive costs. As shown in the previous rules, there are other, more convenient techniques available for handling large scales, for example, avoiding showing an overview of the entire network altogether or choosing an alternative representation (e.g., an adjacency matrix) instead of node-link diagrams.

**Fig 10:**
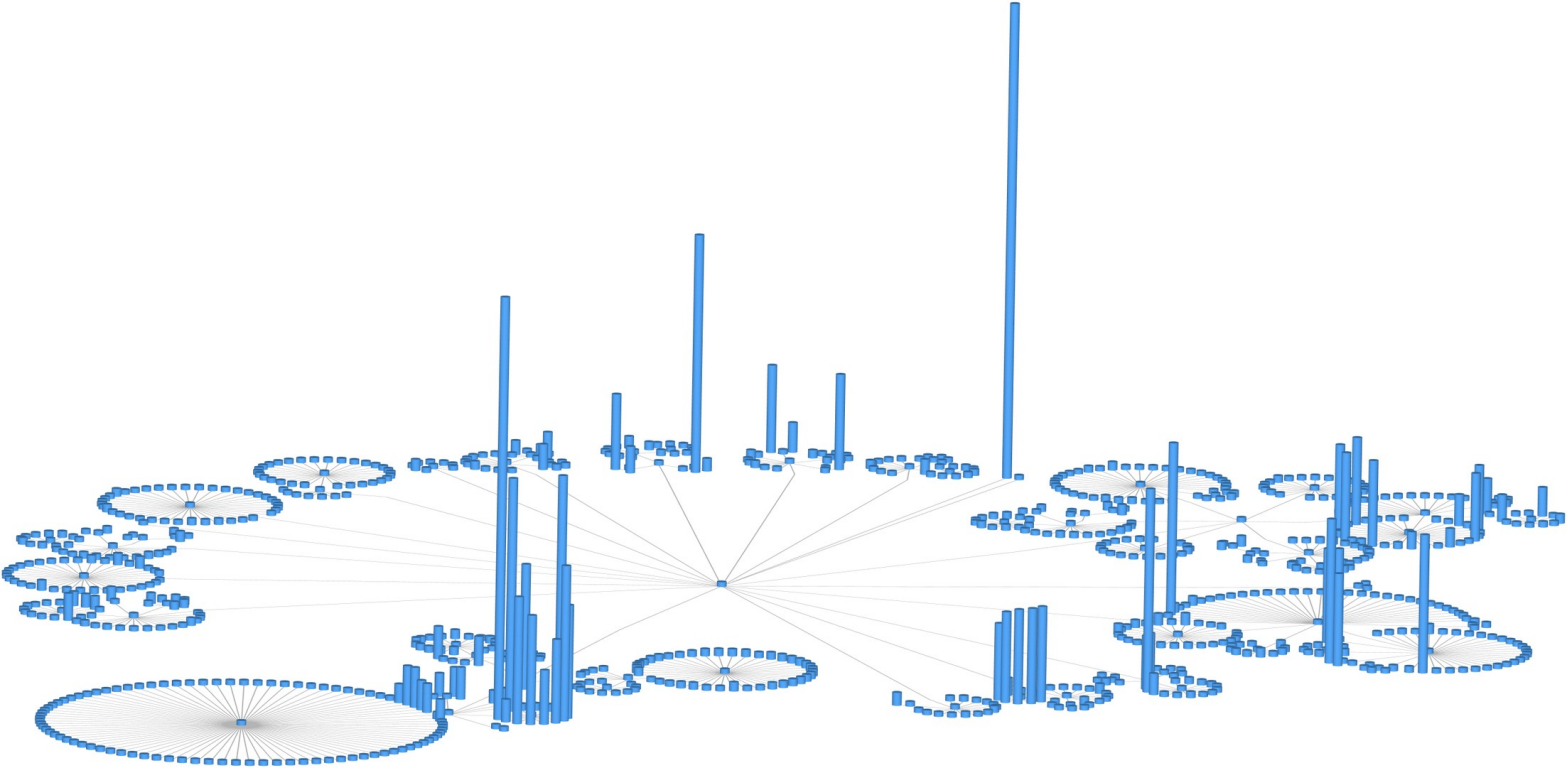
Do not use unjustified 3D. A 2D network displayed along an additional dimension in 3D. The height of each 3D cylinder is mapped to the size of a network attribute. Note the significant number of occlusions. This figure was generated using the open-source software Tulip (see the online Tulip user documentation, Chapter "Tulip in Practice: Four case studies" http://tulip.labri.fr).

The example in Fig 10 shows a network illustration in which the height of each 3D cylinder is mapped to the size of specific network attributes. Note how the different cylinder heights can be mistakenly perceived as perspective foreshortening instead of different attribute sizes. A clearer illustration would use 2D instead and map the attribute size to a visual channel like 2D marker size.

## Conclusion

Several of the examples shown in this paper illustrate the many inherent difficulties in creating biological network figures that are appropriate for communication. The 10 simple rules we outlined in this paper show ways to improve such figures and in several cases, also illustrate the variety of means to visually encode information that circumvent data constraints. We believe these rules will benefit researchers who handle biological networks, be they bioinformaticians, neuroscientists, clinicians, and so on.

We strongly believe that creation of a biological network figure should start with an analysis of the intended figure message (Rule 1). Ideally, this analysis should be performed in conjunction with the domain scientists who generated the network data and its interpretation. Choosing an appropriate basic representation (node-link, matrix, etc.) and layout of the data comes next (Rule 2 and Rule 3), along with the appropriate labels and clarifying text (Rule 4). Gradual data preprocessing through aggregation (Rule 5), appropriate color mappings (Rule 6), the use of an appropriate variety of marks and channels (Rule 7), layering and separation (Rule 8), and sequencing information along several figures (Rule 9) can then help reduce visual clutter and effectively emphasize the message of the figure. With advancements in media technology, we believe 3D figures should be used extremely cautiously, due to documented issues in depth perception (Rule 10).

An important aspect of network visualization that we have shown implicitly, although not discussed directly, is the power of network images to support the integration of a wide variety of data and to encode that data in a number of ways (for example, mapping expression fold change onto node fill color). This is an important and powerful feature of network visualization, particularly for exploring the results of multiple experiments in a single visualization in order to find new hypotheses or to confirm hypotheses, as often done in environments such as Cytoscape. On the other hand, too much information mapped onto a single figure can obscure the key aspects of that figure (see Rule 9), so it is important to balance how much of the network image is about the topology of the network and how much is about the integration of other -omics results in the context of gene or protein relationships. Fittingly, this observation rounds back the discussion to Rule 1—we first need to determine the purpose of the figure.

Another important aspect of network visualization that we have implicitly discussed is the issue of subnetworks. Whereas our rules suggest providing less detail at the periphery of a network, a periphery subnetwork may still be of major interest. Such situations may be addressed through the careful application of Rule 1 (determine first the message of the figure), Rule 8 (use layering and separation) to emphasize the subnetwork, and if necessary, Rule 9 (use multiple figures) to allocate a separate figure to that subnetwork.

Many of the illustrations in this manuscript have been generated using the open-source software platform Cytoscape. Wherever possible, we provided references to the software packages, as well as specific instructions. In an effort to make the application of these rules more accessible, we also provide, wherever possible, the session files for generating these images in a public repository (http://github.com/uic-evl/10RulesBionets). Whereas obviously there are many other software tools for network visualizations, we hope that knowing how to implement these rules in one tool might help the reader more easily transfer that knowledge to another tool. Beyond the basic "how-to" mechanics of the rules, we further that recommend biology researchers contact the biological data visualization community (e.g., http://biovis.net, http://bivi.co, http://visguides.org) for expert advice and help.

We trust that this minimal set of rules helps demystify the process of creating quality static biological network illustrations for communication. Although the landscape of visualization design is far more complex than briefly discussed in this paper, we hope this discussion clarifies some of the most common issues that arise in the creation of network figures, along with basic guidelines to help address those issues. We hope the interested reader will pursue the additional resources and references we include under each rule.
